# Reactive Oxygen Species: Angels and Demons in the Life of a Neuron

**DOI:** 10.3390/neurosci3010011

**Published:** 2022-03-16

**Authors:** Kasturi Biswas, Kellianne Alexander, Michael M. Francis

**Affiliations:** 1 Department of Neurobiology, University of Massachusetts Chan Medical School, Worcester, MA 01605, USA; kasturi.biswas@umassmed.edu (K.B.); kellianne.alexander@umassmed.edu (K.A.); 2 Graduate Program in Neuroscience, Morningside Graduate School of Biomedical Sciences, UMass Chan Medical School, Worcester, MA 01605, USA

**Keywords:** synapse, oxidative stress, *C. elegans*, neurodevelopment, neurodegenerative disease, brain injury

## Abstract

Reactive oxygen species (ROS) have emerged as regulators of key processes supporting neuronal growth, function, and plasticity across lifespan. At normal physiological levels, ROS perform important roles as secondary messengers in diverse molecular processes such as regulating neuronal differentiation, polarization, synapse maturation, and neurotransmission. In contrast, high levels of ROS are toxic and can ultimately lead to cell death. Excitable cells, such as neurons, often require high levels of metabolic activity to perform their functions. As a consequence, these cells are more likely to produce high levels of ROS, potentially enhancing their susceptibility to oxidative damage. In addition, because neurons are generally post-mitotic, they may be subject to accumulating oxidative damage. Thus, maintaining tight control over ROS concentration in the nervous system is essential for proper neuronal development and function. We are developing a more complete understanding of the cellular and molecular mechanisms for control of ROS in these processes. This review focuses on ROS regulation of the developmental and functional properties of neurons, highlighting recent in vivo studies. We also discuss the current evidence linking oxidative damage to pathological conditions associated with neurodevelopmental and neurodegenerative disorders.

## 1. Introduction

Oxygen-derived free radicals, such as the superoxide anion (O_2_^•−^) and hydroxyl radical (^•^OH), as well as non-radical molecules, such as hydrogen peroxide (H_2_O_2_), are collectively known as reactive oxygen species (ROS) [1,2]. ROS are produced as metabolic by-products in different subcellular locations [1,3], including mitochondria [4], endoplasmic reticulum [5], peroxisome [6], plasma membrane [7] and cytosol [8]. Exposure to pathogens, harmful chemicals, heat, UV radiation [9], and heavy metals [10] that potentially damage mitochondria can induce cycles of ROS production and increase cellular ROS concentration. ROS are highly reactive and have the capacity to interact with different biomolecules. At high concentration, ROS can damage DNA, protein, and lipid and disrupt the plasma membrane [1,11], ultimately leading to cell death [12]. Oxidative damage is implicated in several pathological conditions, including major neurodegenerative diseases such as Alzheimer’s and Parkinson’s disease [13], muscular dystrophy [14], and other disease conditions such as chronic inflammation and tissue injury [15], diabetes mellitus [16], and cancer [17]. Further, toxic oxidative metabolites can potentiate necrosis and apoptosis following neuronal injury [18]. To protect from the deleterious effects of oxidative damage, healthy cells need to be equipped with dedicated protective mechanisms such as antioxidant synthesis. Antioxidants can be either enzymatic (superoxide dismutase, catalase, glutathione peroxidases) or nonenzymatic biochemicals (flavonoid, ascorbic acid, tocopherol, etc.) [19]. When antioxidants fail to maintain the cellular ROS concentration within an appropriate physiological range, oxidative stress occurs.

### Controlled Synthesis of ROS and Its Physiological Importance

When acting at appropriately controlled physiological levels, ROS serve important roles as signaling molecules. Indeed, evolutionarily conserved enzymes are dedicated to ROS synthesis. Phagocytic NADPH oxidase (Phox), the first enzyme found to anabolize ROS, was discovered over sixty years ago [20]. Since this time, well over 50 ROS generating enzymes have been identified in human cells [21]. Transmembrane protein NADPH oxidase (nicotinamide adenine dinucleotide phosphate oxidase) serves as the major anabolic source of ROS. In particular, the human genome encodes seven catalytic components of the NADPH oxidase (nicotinamide adenine dinucleotide phosphate oxidase) complex, i.e., NOX 1-5 and DUOX1-2 [20,21,22]. The NOX family enzymes, such as NOX2, reduce oxygen to superoxide [20] via transmembrane electron transport. NOX-produced superoxide is the source of all ROS (hydrogen peroxide, hydroxy radical, etc.) in the phagosome and, by killing invading pathogens, ROS serve essential roles in host defense [23]. Dual oxidases (DUOX1-2) reduce extracellular oxygen by transferring electrons from intracellular NADPH and synthesizing hydrogen peroxide (H_2_O_2_), a non-radical form of ROS [24]. Superoxide is also catabolized by the major antioxidant enzyme, superoxide dismutase, to produce H_2_O_2_. H_2_O_2_ is an important second messenger in various cellular signaling pathways [25]. For example, H_2_O_2_ inhibits phosphatase activity to regulate tyrosine phosphorylation during insulin signaling [26,27], activates MAP kinases in endothelial cells [28], and regulates ion channels and Ca^2+^ signaling in neurons [29].

**Figure 1:**
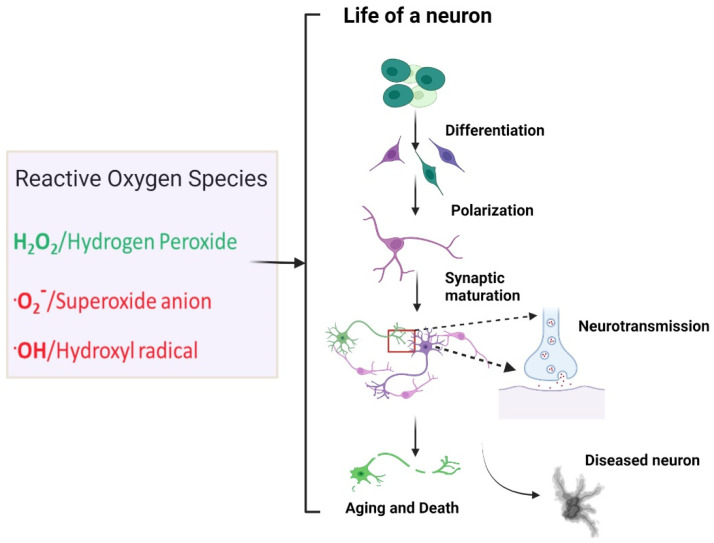
Influence of reactive oxygen species in the life of a neuron. Under normal conditions, the diffusible nonradical ROS, hydrogen peroxide, is synthesized physiologically and is a key regulator of processes fundamental to neuronal development and function, including neuronal differentiation, polarization, synaptic maturation, neuropeptide secretion, and neurotransmitter receptor transport. On the other hand, uncontrolled ROS production by damaged mitochondria, especially of superoxide and hydroxyl radicals, are potentially detrimental. Uncontrolled ROS may damage neurons, promote neuronal decline, and exacerbate neurodegenerative pathology. Created with BioRender.com (accessed on 17 February 2022).

In the next section, we will focus on the roles that ROS play in neuronal development and function. NOX enzymes are highly expressed in neurons, glia (microglia, astrocytes), and neurovascular tissues [30]. In particular, ROS have crucial signaling roles in processes underlying neuronal development and neural circuit assembly. This poses the following interesting question: how do ROS manage to affect the life of a neuron in both positive and negative ways? The studies outlined in this review suggest that ROS have specific roles in either neuronal development and function or neuronal death depending on the nature of ROS, ROS concentration, site of action, and age of the organism (Figure 1).

## 2. ROS in Neurodevelopment

ROS signaling is an important regulator of neuronal differentiation and polarization, axon outgrowth, synapse formation, and synapse maturation [3,25]. In the following sections, we explore the relationship between ROS signaling and neurodevelopment with discussion of examples from both in vitro and in vivo studies.

### 2.1. ROS in Neurogenesis and Differentiation

Neurons are generated from neural stem cells and neuronal progenitors during neurogenesis. NADPH oxidases, particularly NOX2, are expressed in the developing brain during embryogenesis [25,31]. Cortical progenitors and progenitor-derived neurons actively synthesize ROS [32], suggesting that ROS may perform signaling roles in early neurogenesis. Consistent with this idea, ROS levels influence the timing of neurogenesis in the murine embryo [33]. Neuronal differentiation from primary neuronal progenitor cells and the establishment of neuronal identity depend on a complex array of biochemical interactions between growth factors such as nerve growth factor (NGF) [34] and other molecular regulators. NGF treatment triggers an increase in ROS in cultured PC12 cells. Prevention of ROS production inhibits NGF-triggered differentiation of PC12 cells, indicating that ROS may have important intracellular signaling roles during neuronal differentiation [35]. Additional evidence for ROS regulation of neuronal differentiation comes from studies of Neuregulins, a large family of EGF-like signaling molecules that are highly expressed in the nervous system. Neuregulin activation of ErbB receptor tyrosine kinases induces neurite outgrowth in PC12 cells through activation of the MAP kinase signaling pathway [36]. Interestingly, neuregulin treatment was also found to increase intracellular ROS. Treatment with a ROS scavenger inhibited both neuregulin-induced Ras and ERK activation and neuronal differentiation, implicating ROS in the molecular regulation of neuregulin-mediated differentiation [37]. Further investigation revealed that the kinetics of cellular ROS production is an important factor in the cellular decision to divide or differentiate [37].

Recent studies also provide evidence that ROS can act as signals for adult neurogenesis in both the central [38] and peripheral nervous systems [39]. Endogenous H_2_O_2_ regulates growth signaling to maintain proliferating adult hippocampal progenitor cells [40]. Similarly, inhibition of ROS biosynthesis retards proliferation and neurogenesis of neural stem cells in the adult newt brain [41]. These reports and additional accumulating evidence argue for a reconsideration of the view that ROS serve primarily deleterious roles in the nervous system and support a new acknowledgment of roles for ROS signaling in both embryonic and adult neurogenesis and differentiation. 

### 2.2. ROS in Neurite Outgrowth and Polarization

The outgrowth of polarized axons and dendrites is a critical step in the development of brain circuits. Interestingly, ROS have emerged as positive regulators of this process. NOX synthesis of H_2_O_2_ can induce axon and dendrite formation and maturation [25]. Inhibition of the NOX complex disrupts the timing of neuronal polarization, shortens axonal length, and alters the actin cytoskeleton of cultured hippocampal neurons [42]. Furthermore, ROS regulate actin cytoskeletal dynamics and control neurite outgrowth in Aplysia neurons [43]. Recent evidence also suggests a potential role for ROS in the process of axonal specification. The polarity of hippocampal neurons is defined by a polarized distribution of evolutionarily conserved polarity proteins, such as mPar3 and mPar6. Phosphatidylinositol 3-kinase pathway (PI3′K) signaling is critical for achieving this polarized distribution [44]. Physiological levels of ROS have been shown to influence PI3′K signaling by inactivating phosphatase and tensin homolog (PTEN) [45]. It will therefore be interesting to investigate how changes in neuronal redox state might impact the subcellular distribution of polarity proteins and the selection of future axons. Normal physiological levels of ROS support neurite outgrowth and potentially axonal specification, but what molecular mechanisms are instrumental for maintaining physiological ROS concentrations in neurons? Recent findings have offered evidence that a novel feedforward mechanism involving NOX-mediated ROS production and intracellular Ca^2+^ signaling may have a central role [46]. Ryanodine receptor (RyR)-mediated Ca^2+^ release from the endoplasmic reticulum promotes axon extension and is supported by NOX-generated ROS. RyR activation also induces H_2_O_2_ production by NOX through a Rac-1 (Rac Family Small GTPase 1) dependent mechanism [46], offering a potential route for feedforward regulation. As our understanding of mechanisms for physiological regulation by ROS continues to evolve, continuing in vivo molecular studies will undoubtedly reveal new facets of the interplay between ROS and developmental regulators. 

### 2.3. ROS Influence Growth Cone Guidance and Synaptic Maturation

Critical steps in the development of the nervous system include the growth of axons to meet their cellular partners and the subsequent formation of specialized connections called synapses. Axon outgrowth is a complex process involving dynamic changes in the neuronal cytoskeleton. These cytoskeletal dynamics are important for guiding the growing tip of the axon (growth cone) to its destination and are regulated by attractive or repulsive cues in the extracellular environment. Semaphorin is a major repulsive cue for axons, and semaphorin signaling can induce the collapse of growth cones [47]. Semaphorin3A (Sema3A) triggers microtubule disassembly through phosphorylation of the cytoskeletal component regulator, collapsin response mediator protein 2 (CRMP2) [48]. Sema3A triggers an increase in H_2_O_2_ in the growth cones of dorsal root ganglion axons through activation of the multidomain redox enzyme Mical. Subsequently, H_2_O_2_ oxidizes CRMP2, enabling phosphorylation by glycogen synthase kinase-3 (GSK-3) and promoting growth cone collapse [49]. Another recent study showed that NOX2 acts downstream of the Slit2/Robo2 signaling pathway during the growth and guidance of retinal ganglionic cell (RGC) axons in the zebrafish embryo [50]. Related studies have implicated ROS in the regulation of the growth of synapses. A genetic screen for mutants with synaptic overgrowth at the larval neuromuscular junction of *Drosophila* identified *spinster* mutants [51]. The causal mutation in *spinster* is a loss of function mutation in a putative lysosomal efflux permease. Lysosomal dysfunction in *spinster* mutants leads to an increased ROS burden. Interestingly, reduction of ROS in *spinster* mutants normalized the synaptic overgrowth phenotype, suggesting a link between synaptic overgrowth and oxidative stress [52]. Further work implicated ROS activation of the JNK pathway in synaptic growth [52].

Taken together, the findings described in these studies support an expanded view of physiological ROS regulation of key neurodevelopmental events, including axon guidance, synapse formation, and synapse maturation.

### 2.4. Mitochondrial ROS Facilitate Synaptic Pruning by Intrinsic Apoptosis

In most nervous systems, there is an excess of synaptic connections during early development compared with at maturity. Juvenile connections often undergo a refinement process called synaptic pruning, in which weaker or inappropriate connections are eliminated to generate the mature circuit [53,54]. Significant questions remain about the molecular pathways that identify such synapses and initiate their elimination. Mitochondrial ROS are emerging as potential cell-intrinsic factors important for synapse elimination [55,56]. Recent studies of motor behavior in *Xenopus* tadpoles suggest a regulatory role for mitochondrial ROS in synaptic pruning at the neuromuscular junction (NMJ) [56]. The authors found that forced synaptic inactivity increased mitochondrial ROS and mitochondria-targeted antioxidants reduced motor deficits associated with endogenous pruning. Interestingly, this model challenges the prevalent idea that increased neuronal activity is linked with an increase in mitochondrial ROS generation [57,58]. A follow-up communication from this group provided a hypothetical model accounting for their findings, which suggests that neuronal activity may mask a cue for pruning by suppressing mitochondrial ROS production [55]. Mitochondrial O_2_^−^/H_2_O_2_ concentration would therefore surpass the pruning threshold only at inactive synapses, thereby locally activating intrinsic apoptotic cell death signaling pathways to initiate synapse elimination or pruning [55]. Importantly, intrinsic apoptosis can be initiated, propagated and amplified by mitochondrial ROS [59,60,61]. Related studies of *C. elegans* neurons have shown that elimination of presynaptic material involves axonal mitochondria and apoptotic signaling [62], though specific roles for ROS in this process were not determined. An enhanced understanding of the molecular mechanisms that relate neuronal activity to ROS generation and their influence on the stability of synapses will perhaps emerge from ongoing investigations in this area.

### 2.5. Oxidative Damage in Neurodevelopmental Diseases

Interestingly, while physiological ROS have emerged as positive regulators of the processes underlying neuronal development, mounting evidence has also linked oxidative stress and damage with the pathophysiology of neurodevelopmental and neuropsychiatric diseases. Autism spectrum disorders (ASD) are a heterogeneous group of neurodevelopmental abnormalities that manifest as social and cognitive impairments in children and young adults. Post-mortem brain samples from temporal cortices and cerebella of autistic subjects showed a decrease in antioxidants such as glutathione (GSH) and reduced GSH/GSSG redox/antioxidant capacity [63]. Additionally, red blood cells of autistic children have altered antioxidant (SOD, catalase, and GSH) levels compared to controls [64,65]. For a detailed description of oxidative stress in ASD, readers should refer to the review by Pangrazzi [66]. Oxidative stress is also thought to influence the progression of disease pathology in schizophrenia patients [67,68]. Proteomic and metabolomic analyses of superior temporal gyrus and prefrontal cortex tissues from schizophrenic individuals revealed mitochondrial dysregulation, compromised brain metabolism, and oxidative stress [69,70]. Intriguingly, low levels of antioxidant enzymes such as GSH have also been reported in blood samples from patients with schizophrenia [67,71,72]. Because enzymatic activity remained unchanged [73], this result is most consistent with a reduction in GSH synthesis by antioxidant producing cells. Malondialdehyde (MDA) is generated by the peroxidation of membrane polyunsaturated fatty acids and is commonly used as a biomarker to assess oxidative stress [74]. High MDA levels have been reported in adult attention-deficit hyperactivity disorder (A-ADHD), which may point toward an association with oxidative stress [75,76,77]. Consistent with this, increased levels of MDA and decreased levels of the MDA catabolic enzymes paraoxonase and arylesterase were reported in patient serum. However, no correlation between these molecular parameters and disease severity was found [77]. Most studies investigating oxidative stress in the context of neurodevelopmental disease are heavily reliant on analysis of post-mortem tissue or blood serum. It is therefore difficult to assess causality from these analyses. Is oxidative damage a result of, or a cause of, neurodevelopmental abnormalities? Further investigation into important questions surrounding how ROS can both positively and negatively affect neurodevelopment will be critical for teasing apart this central problem of cause and effect. Future exploration of the molecular basis of oxidative damage in neurodevelopmental diseases may also help to identify novel drug targets and potential therapies.

## 3. Roles for ROS in Mature Neurons

### 3.1. ROS in Synaptic Plasticity

There is mounting evidence that reactive oxygen species contribute toward regulation of core neuronal functions such as neurotransmission and synaptic plasticity. Synaptic plasticity is the cellular foundation for learning and memory and refers to structural and molecular modifications at synapses that influence the strength of communication between neurons [78]. The cross-talk between ROS, Ca^2+^ influx, and age-related deficits in the synaptic plasticity of hippocampal neurons has been extensively reviewed elsewhere [79]. Likewise, there is a wide literature available exploring the link between ROS, synaptic plasticity, and memory [80,81,82]. Here we focus our discussion on several recent in vivo studies investigating the physiological regulation of synaptic plasticity by ROS. In *Drosophila* larvae, neuronal ROS were found to be instrumental for neuronal activity dependent structural plasticity of both pre and postsynaptic terminals [58]. Moreover, embryonic and larval motor neurons of *Drosophila* use ROS as key messengers in dendritic plasticity. For example, a recent *Drosophila* study showed that neuronal activity triggered extracellular H_2_O_2_ synthesis by Dual Oxidase. ROS entry into the neuron required neuronal aquaporin expression and was required for structural changes in dendritic arbors [83]. Recent work from *C. elegans* showed that intracellular ROS can modulate the transport and synaptic localization of AMPA-type glutamate receptors through regulation of neuronal Ca^2+^ signaling [84]. This study suggests a mechanistic link between physiological ROS, Ca^2+^ signaling, and excitatory glutamate neurotransmission. Further, studies in hippocampal neurons showed that strong Ca^2+^ transients prolong the lifetime of phosphorylated CREB, a key molecule involved in long-term memory, through enhanced mitochondrial super oxide production [85]. Changes in neuronal oxidative state may also alter Ca^2+^ signaling events that influence hippocampal memory formation [80]. Collectively, the available literature suggests that ROS regulate synaptic plasticity and memory formation in a Ca^2+^ dependent manner.

### 3.2. ROS Influence Neurotransmission

Redox influences on neurotransmission have been described for small-molecule neurotransmitters. A pioneering study at the lobster neuromuscular junction showed that H_2_O_2_ exposure decreased release of the excitatory neurotransmitter glutamate [86]. There are numerous reports of ROS effects on GABA-mediated inhibitory neurotransmission and these have been extensively reviewed [87]. In particular, patch clamp recordings from cultured mouse hippocampal neurons and CA1 pyramidal neurons in hippocampal slices showed H_2_O_2_ directly modulates GABA_A_ receptor function [88]. Studies of the frog neuromuscular junction showed that exposure to Zn^2+^ and Cd^2+^ enhances mitochondrial ROS levels, leading to desynchronization of cholinergic neurotransmitter release [89]. This effect was ameliorated by antioxidant treatment. More recent *C. elegans* studies have provided evidence that axonal mitochondria and ROS production can also modulate neuropeptide release. Blocking mitochondrial transport into axons or disrupting oxidative phosphorylation inhibited neuropeptide release [90]. In addition, increases in endogenously produced H_2_O_2_ from axonal mitochondria were shown to enhance neuropeptide secretion [91]. Changes in diet were sufficient to trigger rapid alterations in endogenous ROS. Elevated neuropeptide secretion in response to endogenous ROS triggered transcriptional activation of antioxidant response genes, offering a potential mechanism for neural control of oxidative stress responses. These studies point toward a role for ROS as secondary messengers that promote neuropeptide secretion.

While there is an increasing appreciation that ROS-mediated signaling plays a positive role in the regulation of neuronal development and function, abundant evidence also links ROS dysregulation with aging neurons and neurodegenerative disease.

## 4. The Dark Side of ROS in the Aging Brain

ROS accumulation in the aging nervous system can have detrimental effects. The management of ROS in the nervous system depends on the timing of ROS production, cellular location, and concentration [25]. The brain accounts for only 2% of human body weight, but utilizes more than 20% of the total metabolic energy [92,93,94]. Maintaining the ionic balance across neuronal membranes, neurotransmission, and protein trafficking are all metabolically demanding processes. A typical glutamatergic neuron may utilize as much as 1.64 × 10^5^ ATP per glutamate-filled vesicle that is released, and the energy cost for one action potential is estimated to be 7.1 × 10^8^ ATP/neuron/spike [95]. ROS are unavoidable by-products of ATP synthesis during aerobic metabolism, produced through premature electron leakage from the mitochondrial electron transport chain (ETC) complex, resulting in an incomplete reduction of oxygen [96]. In a quiescent state, 0.2% of the total oxygen consumed during mitochondrial respiration is converted into superoxide [97]. Ferrous ions are abundant in the brain and are another factor that may serve to increase the load of potentially harmful ROS in the brain. Ferrous ions can promote the conversion of non-radical hydrogen peroxide into hydroxyl radicals through the Fenton reaction [98]. Intriguingly, though ROS produced by mitochondria are often viewed as the villain in promoting neuronal decline with aging, one interesting study suggests neuronal mitochondria are victims of ROS produced by NOX, in particular NOX4 [99]. This in-vitro study showed NOX4 is expressed in neuronal mitochondria and produces superoxide [99]. Additionally, NOX4 was found to inhibit ETC complex 1 [100]; this might further increase ROS production during ATP synthesis. In the following section, we will explore the controversial relationship between aging and oxidative stress.

### 4.1. Oxidative Stress in Aging

A major theory of aging, termed the free radical theory (FRTA), regards ROS accumulation as one of the earliest hallmarks of aging [101]. According to this theory, mitochondrial dysfunction is a primary cause of global cellular damage and neuronal aging. As cells age, the efficacy of the electron transport chain decreases [102], leading to premature leakage of electrons during ATP synthesis and a potential increase in the cellular concentration of ROS. Though it is clear that high levels of ROS are toxic, the roles of ROS in aging are complex and remain controversial. Numerous studies across yeast, flies, and mice have highlighted the importance of antioxidant defense mechanisms. In particular, mutation of the major antioxidant *sod* genes, especially mitochondrial *sod-2* in yeast [103,104,105], flies [106,107], and mice [108,109] severely reduce lifespan. However, over time, the role of ROS in aging has been challenged. Seminal work in *C. elegans* showed that deletion of the same mitochondrial antioxidant *sod-2* extends lifespan [110,111,112] and knockout of all *sod* genes, and thus SOD activity in *C. elegans* resulted in normal longevity [113]. Similarly, mice that lack one copy of Sod2 (Sod2^+/−^) show prolonged lifespan compared to wild-type animals despite having enhanced mitochondrial oxidative stress [114]. Additionally, elevated mitochondrial ROS failed to accelerate aging in mice [114,115]. Conversely, mutation of the mitochondrial DNA polymerase accelerated aging in mice without any increase in oxidative stress markers [116,117]. Together, these results challenge the long standing FRTA theory. Interestingly, mild mitochondrial stress triggers cytoprotective pathways such as the mitochondrial unfolded protein response (mitoUPR) that may render animals less susceptible to subsequent physiological perturbations. This phenomenon is termed mitohormesis [118,119]. Activation of the mitoUPR was shown to promote the longevity of *C. elegans* mutants with impaired mitochondrial respiration, such as *isp-1* and *clk-1* mutants [120] (*clk-1*/human ortholog of coenzyme Q7 and *isp-1*/human ortholog of ubiquinol cytochrome c reductase). These mutants live longer [121,122] despite increased levels of ROS [123]. Organismal longevity may therefore reflect an integration of the damaging effects of ROS with the activation of pro-survival pathways through processes such as mitohormesis. It will be interesting to investigate the extent to which mitohormesis contributes to ROS regulation of neuronal development and function.

A revised view of aging argues that mitochondrial ROS are unlikely to be a primary cause but may act in combination with diverse epigenetic factors and a failure of quality control mechanisms to regulate the rate of aging in the brain [117]. Neurons are non-dividing cells [124], potentially accruing high levels of ROS over time and as a consequence are potentially susceptible to protein accumulation and DNA damage [125]. Additionally, oxidative stress is regarded as a risk factor in neurodegenerative diseases including Alzheimer’s disease, Parkinson’s disease, Huntington’s disease, and amyloid lateral sclerosis [13,126]. Below we briefly discuss evidence for involvement of oxidative damage in each.

### 4.2. Oxidative Damage in Neurodegenerative Disorders 

Physiologically, aging is characterized by a heightened sensitivity to environmental stress. External stressors, such as pathogens [127], heat [128] and radiation [129] can all lead to mitochondrial damage. Mitochondrial injury can trigger a damaging cycle of ROS production where oxidative stress exacerbates additional cellular pathologies associated with neurodegenerative disease [126]. The role of oxidative damage is widely discussed in various models of Alzheimer’s disease (AD) [130]. A major pathological hallmark of AD is the formation of amyloid β (Aβ) plaques. Aβ was shown to accumulate in mitochondria and impact mitochondrial function prior to the development of widespread extracellular Aβ deposits [131]. Genetic and pharmacological manipulations that decrease mitochondrial superoxide have been shown to reverse Aβ-induced impairment of long-term potentiation in a mouse model of AD, implicating imbalances of mitochondrial ROS in the Aβ-induced disruption of hippocampal synaptic plasticity [132]. ROS imbalances are also associated with the occurrence of motor deficits, such as resting tremor and rigidity, that are commonly observed symptoms during the progression of Parkinson’s disease (PD). Evidence for oxidative damage in PD has been comprehensively reviewed by Dias and colleagues [133]. Progressive degeneration of dopaminergic neurons in the substantia nigra (SN) is a major pathological hallmark of PD [134,135], and ROS-mediated damage to dopaminergic neurons is observed in both animal models and post-mortem brains [135]. Studies of post-mortem samples from the brains of PD patients revealed increased levels of ROS-modified biomolecules such as peroxidized lipid and the presence of protein oxidative damage in the form of carbonylated proteins [136]. NOX1 activity has been shown to increase α-synuclein expression and aggregation in both cultures of human dopaminergic neurons and rat models of PD [137], suggesting a contribution of enzymatically synthesized ROS to PD pathology. Oxidative damage is also evident in cases of Huntington’s disease (HD) [138,139], another severe neurodegenerative disorder. An autosomal dominant mutation of the huntingtin (*htt*) gene produces an abnormal CAG repeat expansion and gives rise to characteristic symptoms of HD such as involuntary muscle movement and muscle dystonia [140]. As HD arises from modification of a single genetic locus, HD models are powerful for understanding how environmental stressors, such as ROS, may affect the progression of neurodegenerative pathophysiology. Interestingly, HD brain samples show elevated levels of the antioxidant enzymes SOD (Zn/Cu-SOD and mitochondrial MnSOD), glutathione peroxidase, and catalase [141], suggesting an oxidative insult to the system. Finally, mitochondrial oxidative damage has been demonstrated in amyloid lateral sclerosis (ALS) [142], which primarily affects motor neurons. Mutations in the antioxidant enzyme SOD are among the most common mutations in familial forms of ALS and the most widely used transgenic models of ALS [143]. Though unlikely to be the primary cause of motor neuron death, loss of SOD1 function in some instances of familial ALS may result in oxidative damage and contribute to disease progression. In addition, examination of spinal cord tissue from sporadic ALS patients has revealed traces of lipid peroxidation and protein glycoxidation, suggesting a strong association between oxidative damage and disease [144]. 

### 4.3. ROS in Secondary Brain Injury

Acute ischemic stroke induces a rapid increase in ROS in the brain that further exacerbates primary stroke damage [145]. In a rat model of ischemia, increases in cortical ROS levels were observed soon after middle cerebral artery occlusion [146]. A recent study of brain injury following intracerebral hemmorhage (ICH) also provided evidence for ROS involvement. Brain CT scans from patients with acute ICH showed that early and high dosage of ROS scavengers significantly reduced the volume of perihematomal edema [147], a quantifiable marker of secondary brain injury post ICH [148]. 

## 5. Emerging Concepts Linking Redox Biology and Neuroscience

The dual roles of ROS as both key signaling molecules and agents of damage in the nervous system have spurred intense interest from neurobiologists. Further investigation into concentration-dependent effects of ROS and mechanisms for regulation of cellular ROS levels will be critical for understanding how ROS impact neurons under both normal and pathophysiological conditions. Specifically, additional exploration of the diverse molecular regulators that balance ROS, the molecular pathways that maintain the threshold of ROS concentration, and the mechanisms that dictate ROS action in different physiological contexts is required. An improved understanding of neuronal redox biology may help to promote the development of targeted therapies that act through modulation of cell signaling. For example, in a mouse model of AD, dietary supplementation of the mitochondrial antioxidant coenzyme Q reduced brain protein carbonyl levels, a biomarker of oxidative damage [149]. Moreover, developing biomolecular screens to identify novel neuroprotective strategies against ROS has immense therapeutic potential. Recent work has demonstrated that Baicalin, a plant-derived flavonoid, acts via Akt/Nrf2 antioxidant signaling to provide neuroprotection in a mouse model of traumatic brain injury [150].

Accumulating evidence suggests there may be differences in male and female vulnerability to neurodegenerative diseases. For instance, preclinical and clinical evidence suggest that women have higher risks for Alzheimer’s disease than men [151] and female AD patients suffer greater cognitive deterioration [152]. In contrast, the incidence of Parkinson’s disease is almost twice as high in males compared with females [153]. We still lack a molecular understanding of this sexual dimorphism and potential ROS involvement. As ROS affect almost every stage of neurodevelopment, it will be interesting to investigate whether redox modulation divergently influences the structure, function, and vulnerability of male and female nervous systems. Studies of ROS signaling and oxidative damage have typically relied heavily on biomarkers and oxidative stress assays. With the development of fluorescent sensors, we can now directly quantify ROS both in cell culture and in in vivo animal models. Genetically encoded ROS sensors such as HyPerRed [154] and redox-sensitive green fluorescent protein (roGFP) [155] offer significant advantages for investigating how physiological and environmental factors impact the redox environment of specific cell types and organelles. For example, studies using roGFP in *C. elegans* demonstrated that proteotoxic challenges triggered by aging had the opposite effects of the redox environments of the cytosol and endoplasmic reticulum [155]. In vivo quantification of H_2_O_2_ in the zebrafish embryo using HyPer was instrumental in demonstrating that spatiotemporal alterations in H_2_O_2_ regulate axon pathfinding [156]. Further, these sensors may be used in screens to identify novel neuron and glia signaling pathways involved in ROS regulation. Recent technical advances in fluorescent microscopy approaches have enabled the detection of redox alterations at the subcellular level using fluorescent ROS sensors. For example, two-photon fluorescence microscopy has been used to detect UV radiation induced ROS synthesis in epidermis [157]. Furthermore, super resolution or light-sheet microscopy approaches in combination with fluorescent ROS sensors offer immense potential for investigating the relationship between ROS accumulation and protein aggregation in neurodegenerative disease models. Additionally, recent advances in single cell transcriptomics present approaches with high power for investigating genetic factors that potentially contribute to cellular susceptibility to oxidative damage. For example, a recent single neuron RNA-sequencing study showed that an upregulation of mitochondrial respiratory chain complex genes is associated with oxidative damage in a mouse model of Parkinson’s disease [158]. Further, recent transcriptional profiling studies identified glutathione pathway genes as part of an oxidative stress gene signature shared across resident microglia and infiltrating macrophages in a multiple sclerosis model, uncovering a novel molecular signaling network [159]. ROS levels and redox alterations are gaining importance as indicators in therapeutic development, especially in efforts to identify drug candidates. Endogenous contrast MRI has proved useful for detecting brain ROS levels in patients before and after therapeutic interventions to manage oxidative damage [160]. Biosensors that rely on the detection of electroactive H_2_O_2_ produced by enzymatic reactions in the biosensors have recently been developed to detect neuronal release of glutamate and GABA in vivo. A microarray probe consisting of two such biosensors allowed continuous detection of glutamate and GABA in real time from cell culture or brain tissue samples [161]. The simultaneous detection of excitatory and inhibitory neurotransmitters may prove powerful in screens of drug candidates to treat brain disorders, such as epilepsy, associated with imbalances in excitation and inhibition. With the dramatic progress in biotechnology and nanomedicine over the last two decades [162], the effective delivery of ROS scavengers or other neuroprotective elements to ameliorate oxidative damage in the human brain may soon be closer to reality.

## 6. Significance

We have highlighted the context-dependent effects of reactive oxygen species throughout the life of a neuron, summarizing the latest advancements in the field as well as ongoing questions surrounding the roles for ROS signaling in neurodevelopmental processes and potentially destructive roles for ROS during age-related pathologies, neurodegenerative disorders, and cerebrovascular disease. Gaining an improved understanding of how ROS signaling regulates neuronal function and identifying protective molecular mechanisms that work to minimize oxidative damage in neurons will be critical for the continuing efforts to develop new therapeutic approaches to combat conditions associated with oxidative damage in the nervous system.
